# Cardiovascular Disease and Stroke in Immune TTP–Challenges and Opportunities

**DOI:** 10.3390/jcm12185961

**Published:** 2023-09-14

**Authors:** Senthil Sukumar, Marshall A. Mazepa, Shruti Chaturvedi

**Affiliations:** 1 Division of Hematology/Oncology, Department of Medicine, Baylor College of Medicine, Houston, TX 77098, USA; senthil.sukumar@bcm.edu; 2 Division of Hematology, Oncology, and Transplantation, Department of Medicine, University of Minnesota, Minneapolis, MN 55455, USA; mmazepa@umn.edu; 3 Division of Hematology, Department of Medicine, Johns Hopkins University School of Medicine, Baltimore, MD 21287, USA

**Keywords:** TTP, TMA, cardiovascular disease, stroke

## Abstract

Advances in the management of immune thrombotic thrombocytopenic purpura (iTTP) have dramatically improved outcomes of acute TTP episodes, and TTP is now treated as a chronic, relapsing disorder. It is now recognized that iTTP survivors are at high risk for vascular disease, with stroke and myocardial infarction occurring at younger ages than in the general population, and cardiovascular disease is the leading cause of premature death in this population. iTTP appears to have a phenotype of accelerated vascular aging with a particular predilection for cerebral circulation, and stroke is much more common than myocardial infarction. In addition to traditional cardiovascular risk factors, low ADAMTS13 activity during clinical remission may be a risk factor for some of these outcomes, such as stroke. Recent studies also suggest that Black patients, who are disproportionately affected by iTTP in the United States, are at higher risk of adverse cardiovascular outcomes, likely due to multifactorial reasons. Additional research is required to establish the risk factors and mechanisms underlying these complications in order to institute optimal screening strategies and identify interventions to improve outcomes.

## 1. Introduction

Nearly a century ago, Eli Moschowitz described the case of a 16-year-old girl with fever and hemolytic anemia accompanied by progressive neurologic dysfunction resulting in coma and ultimately death, which is now recognized as the first description of thrombotic thrombocytopenic purpura (TTP) [1]. Over the last century, our understanding of this devastating condition has evolved immensely. Severe deficiency of the von Willebrand factor-cleaving protease, ADAMTS13, which leads to the formation of VWF–platelet microthrombi, has been elucidated as the underlying mechanism of TTP [2]. TTP may be congenital due to biallelic pathogenic mutations in ADAMTS13 or due to an antibody against ADAMTS13. This review focuses on immune-mediated TTP (iTTP), which is more common and represents 95% of TTP. Mortality of acute TTP episodes has reduced from >90% to <10% with rapid diagnosis and prompt treatment with plasma exchange and immunosuppression [3,4]. With a growing population of TTP survivors, immune-mediated TTP (iTTP) is recognized as a chronic disorder that requires long-term care [5,6,7,8]. Importantly, iTTP is a thrombotic vascular disorder, and thromboembolic events such as stroke are a leading cause of mortality and morbidity in acute iTTP [9,10]. Even after surviving acute iTTP, survivors have shortened overall survival, with a nearly two-fold increased rate of mortality when compared to an age-, sex-, and race-matched control population [11,12]. Cardiovascular disease is a leading driver of this increase in mortality [11,12]. Recent epidemiological investigations in both congenital and immune TTP reveal a pattern of accelerated vascular disease and early ischemic events, such as stroke and myocardial infarction, that contribute to mortality and morbidity [13,14,15,16,17]. In this review, we discuss the epidemiology of cardiovascular disease in iTTP, potential risk factors and underlying mechanisms, and review opportunities to improve cardiovascular outcomes in this vulnerable population.

## 2. Cardiovascular Involvement in Acute iTTP

Cardiac involvement in acute episodes of iTTP is heterogeneous and underrecognized [10,18]. Cardiac arrest and myocardial infarction are the most common immediate causes of death in iTTP, and autopsy studies commonly show cardiac involvement [9]. Microthrombosis is the most common finding in autopsy studies, which is the most likely mechanism of ischemic cardiac injury in iTTP [19]. In a systematic review of 111 patients with iTTP, Hawkins et al. reported that the most common cardiac symptoms in iTTP were chest pain (11.7%), CHF (9.0%), and syncope (0.9%), and the most frequent clinical cardiac events were myocardial infarction (23.4%), congestive heart failure (15.3%), arrhythmias (9.0%), cardiogenic shock (5.4%), and sudden cardiac death (7.2%). Overall acute MI rates of approximately 5–15% have been reported in acute iTTP, and non-ST segment elevation MI is more common than ST segment elevation MI [20,21]. While a smaller study did not find an association of traditional risk factors with MI in iTTP, a larger analysis from the United States Nationwide Inpatient Sample suggests that older age, smoking, known coronary artery disease, and congestive heart failure are associated with increased risk of MI with acute iTTP, and overt cardiovascular complications are associated with substantially higher in-hospital mortality [21,22]. Even patients without overt symptoms may have cardiac involvement of iTTP evidenced by troponin elevation and electrocardiogram changes [23]. Troponinemia is associated with acute mortality and refractoriness in iTTP [23,24]. Given the association of symptomatic and asymptomatic cardiac involvement with adverse outcomes in acute iTTP [25], and the contribution of cardiac events as a proximate cause of death [10], we suggest that all patients should undergo at least baseline evaluation with cardiac troponin measurement and an electrocardiogram. Patients with confirmed cardiac involvement may benefit from added telemetry and echocardiography during the acute episode [26].

The management of acute MI in iTTP is made more challenging by coexisting thrombocytopenia and drugs such as caplacizumab, which increase bleeding risk and may preclude aggressive antithrombotic measures. Most patients with non-ST elevation MI can be managed with iTTP-directed therapy, along with medical management of MI with beta-blockers and vasodilators, with the consideration of adding antiplatelet therapy once the platelet count increases to over 30–50^9^/L. Antiplatelet therapy appears to be safe in iTTP. In the seminal trial that established plasma exchange as the standard treatment for iTTP [4], all the patients received either aspirin or dipyridamole; however, the safety of aspirin in patients who are also receiving caplacizumab, a novel anti-VWF nanobody approved for acute iTTP, needs to be established [27]. Caplacizumab binds to the A1 domain of VWF, thus preventing VWF platelet interactions and consequently inhibiting the formation of microthrombi; however, as a result of this mechanism of action, caplacizumab also increases bleeding risk. Targeted coronary interventions have not been widely studied in the context of acute iTTP, which is not surprising since iTTP is an extremely rare, high-risk disorder. However, successful percutaneous coronary intervention has been reported in iTTP with acute STEMI [28].

While neurologic symptoms such as headache and altered mental status attributed to microvascular injury are most commonly recognized in acute iTTP, stroke is reported in 5–10% of acute iTTP episodes [7,29]. The pattern of stroke is heterogeneous, affecting different parts of the cerebral circulation, and is multifocal in 30–40% [30]. When a stroke occurs in the context of acute iTTP, the mainstay of treatment is treatment of the iTTP with plasma exchange and immunosuppression. Similar to acute MI, stroke-specific interventions have not been studied widely in the setting of iTTP. Measures such as thrombolysis carry increased bleeding risk in patients with thrombocytopenia, but rare cases of stroke in iTTP treated with thrombolysis have been reported.

## 3. Cardiovascular Disease Burden in Chronic iTTP

### 3.1. Cardiovascular Disease Contributes to Shortened Survival in iTTP Survivors

Recent reports from multiple iTTP registries show that iTTP survivors are at a higher risk of premature death and, rather than acute iTTP relapse, cardiovascular disease is the leading cause of mortality and morbidity (Table 1) [6,11,12]. For example, of 57 patients followed after iTTP diagnosis in the Oklahoma iTTP registry, 19% died over a median follow-up of 7.8 years, which is higher than expected based on age- and sex-matched U.S. or Oklahoma reference populations [6,11]. The majority (64%) of deaths were attributed to cardiovascular and/or cerebrovascular complications, while only 18% of deaths were attributed to iTTP relapse [6]. Subsequently, we compiled a 222-patient cohort from the Johns Hopkins University and Ohio State University, which also had higher all-cause mortality in iTTP survivors compared to age- and sex-matched reference populations [12]. Similar to the Oklahoma registry, cardiovascular disease (27.6%, 8 of 29) and iTTP relapse (27.6%, 8 of 29) were the leading causes of death [12]. Finally, Prevel et al. reported that older (>60 years) iTTP patients in the French iTTP registry had increased short- and long-term mortality [31]. In addition to the iTTP diagnosis itself, traditional cardiovascular risk factors such as male sex, diabetes, tobacco use, malignancy, hypertension, cerebrovascular events, dementia, and COPD were risk factors for one-year mortality among older iTTP survivors [31].

### 3.2. Epidemiology of Stroke and Myocardial Infarction in iTTP Survivors

In a cohort of 137 iTTP survivors followed for a median observation period of 3.08 years, the risk of stroke during clinical remission was increased nearly five-fold (13.1% vs. 2.6%) compared with an age- and sex-matched control population, and this risk was strongly associated with suboptimal ADAMTS13 recovery during clinical remission [15]. Subsequently, we showed that major adverse cardiovascular events (stroke, non-fatal and fatal MI, and cardiac revascularization) occurred in 24% of iTTP survivors followed for a median of 7.6 years [14]. This rate is more than double previously reported high-risk cohorts including populations with known underlying vascular disease or genetic predispositions [32,33]. Notably, stroke was much more common than myocardial infarction (18.2% vs. 6.8%), a pattern that has also been reported in patients with congenital iTTP, who have a much higher burden of stroke compared to cardiac ischemic disease [13,14,15,16,17]. Additionally, neurologic involvement is also more common in acute iTTP. These observations suggest that the brain is particularly vulnerable to TTP-associated (or ADAMTS13 deficiency-associated) vascular injury. Finally, the age at the first MACE event was 1–2 decades younger than the age at the first MACE event in populations without iTTP [34]. These differences are more striking in young female iTTP survivors, who would not typically be considered at high risk for MACE events compared with usual at-risk populations such as older males. For example, age at first stroke event was younger for individuals with iTTP than individuals in the general population without iTTP for both males (56.5 years versus 68.6 years, *p* = 0.031) and females (49.7 years versus 72.9 years, *p* < 0.001). Similarly, the mean age at first myocardial infarction was lower for males (56.5 years versus 65.6 years, *p* < 0.001) and females (53.1 years versus 72.0 years, *p* < 0.001) with iTTP compared with individuals in the general population without iTTP [14]. This pattern of early cerebrovascular disease has also been reported in cTTP, suggesting that TTP or ADAMTS13 deficiency is characterized by a phenotype of accelerated vascular aging.

### 3.3. Silent Cerebral Infarction in iTTP Survivors and Impact on Functional Outcomes

Most recently, a study reported that in a prospectively enrolled cohort of iTTP survivors, 50% demonstrated silent cerebral infarction, which is defined as magnetic resonance imaging (MRI) evidence of brain ischemic infarction without a corresponding neuro-deficit [35]. Silent cerebral infarction was strongly associated with cognitive impairment, including both major and mild cognitive impairment [35]. Given that silent cerebral infarctions are a risk factor for both cognitive impairment [36] and stroke [37] in the general population, it is likely that silent cerebral infarction also contributes to neurocognitive deficits that are a common complaint in TTP survivors (60–80%) [38,39,40,41,42]. Similar to stroke, the rate of silent cerebral infarction in this relatively young iTTP cohort with a median age of 48 years was much higher than in older cohorts of individuals without iTTP, where the prevalence of silent cerebral infarction ranges from approximately 10% to 20% at a mean age of 60–70 years and increases with age [43,44,45,46,47,48,49]. While this was a small study that did not measure serial ADAMTS13 activity for the entire period since iTTP diagnosis, the patients with SCI had lower mean ADAMTS13 activity than those without SCI in the year preceding evaluation, suggesting again that lower remission ADAMTS13 activity contributes to the risk of cerebrovascular disease in iTTP survivors. The findings of this study are also supported by a previous study from Ohio State University and University College London that reported ischemic findings on brain MRI in 9 of 23 patients with iTTP in clinical remission [41]. Silent cerebral infarction is a risk factor for stroke in the general population [37,50] and individuals with sickle cell disease [51]. This observation as well as shared risk factors with stroke, such as increasing age and hypertension, suggest that silent cerebral infarction is on the spectrum of cerebrovascular disease that culminates in stroke. Moreover, the strong association of silent cerebral infarction with cognitive impairment makes it an attractive target for interventions aimed at reducing neurocognitive morbidity in iTTP survivors.

### 3.4. Factors Contributing to Risk of CV Disease in iTTP

**Figure 1:**
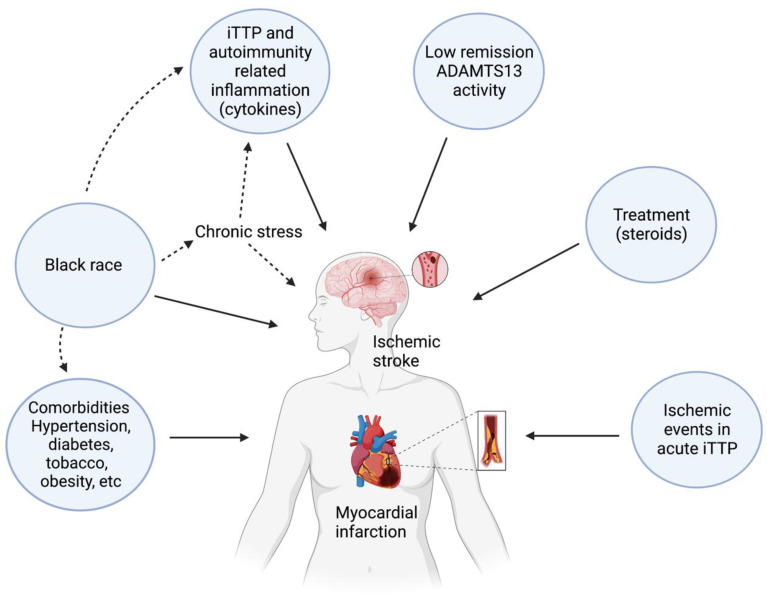
Potential risk factors for cardiovascular disease (stroke and myocardial infarction) in individuals with immune thrombotic thrombocytopenic purpura.

Multiple heterogeneous factors, both related to iTTP as well as other patient characteristics and comorbidities, contribute to the increased risk of cardiovascular disease in iTTP (Figure 1) In a retrospective cohort study from the United States, increasing age and diabetes mellitus were associated with increased rates of MACE in iTTP survivors [14]. This study did not find an association between other traditional risk factors for MACE such as hypertension, obesity, and CKD; however, this may be due to the relatively small sample size. In contrast, a report from the French iTTP registry found that older iTTP patients had 3.4 times higher long-term mortality compared with an age-matched reference population from the same geographic area (a population-based cohort on aging and dementia, the Three City Study) and attributed shortened survival to coexisting disorders, such as hypertension, depression, and cognitive decline [31]. Coupled with the finding that cardiovascular disease is a leading cause of death, these findings suggest that these comorbidities may contribute to lower survival in iTTP survivors. Indeed, compared to a reference population, iTTP survivors have higher rates of comorbidities such as autoimmune disorders [6], obesity [6], hypertension [6], and depression, which are recognized as predictors of all-cause and cardiovascular mortality [52]. The factors driving the higher prevalence of these comorbidities in iTTP are complex and incompletely understood. iTTP is associated with depression and other mood disorders, which are independent risk factors for obesity [53], which may in turn increase cardiovascular morbidity [54]. Obesity is also a risk factor for hypertension, cardiovascular disease, and mortality [55,56]. iTTP more commonly affects women and Black people, the same patient demographic susceptible to autoimmune conditions like SLE [57,58]. In the single study that examined risk factors for major adverse cardiovascular events in iTTP in the United States, Black patients had a 2.3 times higher hazard of adverse cardiovascular events than White patients with iTTP [14]. However, race is not simply a biological construct and is often a surrogate for socioeconomic status, education level, access to resources, and other social determinants of health that have marked impacts on the risk of chronic disease development, as well as overall survival [59,60]. These factors likely also contribute to the increased risk of relapse in Black TTP survivors, which may in turn increase their risk for cardiovascular events. Recent research links racism and discrimination to chronic stress [61], which can have an independent effect on promoting autoimmunity and inflammation [62,63].

One of the more provocative findings from observational studies is the association of lower remission ADAMTS13 activity in iTTP survivors with a number of adverse health outcomes, including stroke [15], silent cerebral infarction [35], and a trend towards higher all-cause mortality [12]. Thus, patients with both immune and congenital TTP, who share a phenotype of partial or complete ADAMTS13 deficiency, develop accelerated vascular aging and atherosclerosis that predominantly affects the cerebral circulation. The rate of vascular events, particularly stroke, is higher than expected based on traditional risk factors. In this setting, remission ADAMTS13 activity is an attractive biomarker to explain the additional risk. This premise is also supported by large population-based cohort studies from the Netherlands, where lower ADAMTS13 activity has been identified as a risk factor for coronary heart disease, stroke, and all-cause and cardiovascular mortality [64,65,66]. The potential mechanism for these findings is that lower ADAMTS13 levels lead to an accumulation of larger, more physiologically active von Willebrand factor multimers that promote platelet activation [67], complement activation [68,69], and accelerate atherosclerosis [65,66,70,71]. This may be pronounced among iTTP survivors, who often do not fully recover ADAMTS13 activity during clinical remission (dubbed partial or incomplete ADAMTS13 remission) [64,72].

### 3.5. Future Directions—Opportunities to Improve Cardiovascular and Neurologic Outcomes

The pathobiology and risk factors for cerebrovascular disease and cardiac ischemia are diverse, and thus potential therapeutic avenues are also diverse and will depend on the phase of the disease (acute iTTP versus remission) and an individual patient’s risk factors. During acute iTTP, microthrombi due to iTTP itself are the main cause of ischemic injury to the brain, heart, and other organs. Cardiovascular assessment and the management of acute MI or stroke during acute iTTP have been discussed above. Novel agents such as caplacizumab that target VWF and theoretically reduce microthrombi formation have been developed and are approved for use in acute iTTP [73]. While clinical trials of caplacizumab did not specifically examine ischemic events as a primary outcome, the Phase 3 HERCULES trial did show that normalization of markers associated with organ damage (including cardiac troponin I) occurred sooner among patients who received caplacizumab than among those who received a placebo [73]. Whether caplacizumab reduces clinically significant cardiac or cerebral ischemia has not been evaluated. It is plausible that the ischemic insults of acute iTTP may be reduced by the prompt use of therapies targeting microthrombi, such as the anti-VWF nanobody, caplacizumab (and novel agents under development such as Microlyse, a VWF-targeting thrombolytic fusion protein) [74]. Analogous to the use of thrombolytics in acute stroke and myocardial infarction, these VWF/microthrombi-targeting drugs may not prevent all ischemic injury but are likely to reduce it, which still needs to be shown in clinical studies.

Remission ADAMTS13 activity is also an attractive therapeutic target because of its association with stroke and other vascular diseases, and the availability and long experience with immunosuppressive agents that target anti-ADAMTS13 antibodies and can help increase ADAMTS13 activity levels. Current iTTP management strategies use preemptive rituximab during ADAMTS13 relapse to target ADAMTS13 activity > 10–20% to prevent relapse [75]. However, higher ADAMTS13-target activity might mitigate iTTP-related cardiovascular morbidity, which needs to be studied and the optimal ADAMTS13 target needs to be established. Antiplatelet or antithrombotic therapy are also attractive approaches to reduce cardiovascular morbidity but have not been specifically studied in iTTP. Until more data on these approaches are available, it is reasonable to screen for and modify any cardiovascular risk factors.

**Table 1:** Summary of studies reporting cardiovascular outcomes in iTTP in clinical remission.

Study	Site (s)	N	Female Sex	Race	Median Age (Years)	Median Follow-Up	Cardiovascular Outcomes
Deford 2013 [6]	Oklahoma Registry	57	79%	White: 63% Black: 37%	39 (range 9–71)	7.8 years	19% mortality
Upreti 2019 [15]	Johns Hopkins Registry	137	67.9%	White: 38% Black: 62%	48.8 (IQR 35.3, 60.3)	3.08 years	Stroke during remission in 13.1%
Brodsky 2021 [14]	Ohio State University and Johns Hopkins Registries	181	71.3%	White: 45.9% Black: 53% Other: 1.1%	39 (IQR 27–51)	7.6 years	23.7% MACE rate in clinical remission.
Sukumar 2022 [12]	Ohio State University and Johns Hopkins Registries	222	70.3%	White: 46.8% Black: 50.5% Other: 2.7%	42 (IQR 29–55)	4.5 years	Mortality 222.8 per 100 patient-years (1.8 times higher than age and sex-matched control cohort). Cardiovascular disease and iTTP relapse (27.6% each) were leading causes of death.
Chaturvedi 2023 [35]	Neurologic Sequelae of iTTP (NeST) Study, Johns Hopkins	36	64.3%	White: 14.3% Black: 66.7% Other: 4.8%	48 (IQR 34–56)	5.5 (IQR 1.5–9.3)	50% had silent cerebral infarction on brain MRI

TTP—thrombotic thrombocytopenic purpura. MACE—Major adverse cardiovascular events (any myocardial infarction, stroke, cardiac revascularization). SCI—silent cerebral infarction.

Our current clinical practice is to pursue aggressive cardiovascular risk factor management by (1) screening for and optimizing management of common risk factors like hypertension and hyperlipidemia, including referral to the appropriate specialists as needed, (2) starting aspirin in patients with low bleeding risk who have known coronary artery disease or peripheral vascular disease, history of stroke (including stroke during acute iTTP), are active smokers, have diabetes mellitus, lupus, or a >10% estimated risk of cardiovascular disease at 10 years, though traditional risk calculators may underestimate risk in iTTP. There are currently no data to guide whether all patients with iTTP should undergo additional tests such as cardiac echocardiography, MRI, or brain MRI in clinical remission, and our practice is to do these studies only when clinically indicated or in the research setting. Ultimately, additional research is required to establish the risk factors and mechanisms underlying long-term cardiovascular complications in iTTP and to establish optimal screening strategies and interventions to improve outcomes that can be tested in clinical trials. Future clinical trials of novel agents for iTTP should also evaluate short- and long-term organ damage that is clinically relevant. Given the rarity of iTTP, this will require multicenter studies, with national and international collaboration, and the support and collaboration of patient advocacy groups.

## Data Availability

No primary data were generated for this manuscript.
